# The impact of banking uncertainty on firm investment: A look into intangible assets

**DOI:** 10.1371/journal.pone.0340913

**Published:** 2026-01-23

**Authors:** Japan Huynh, Thi Minh Hue Phan

**Affiliations:** Faculty of Finance and Banking, Ho Chi Minh City Open University, Ho Chi Minh City, Vietnam; Chosun University, KOREA, REPUBLIC OF

## Abstract

This study examines the impact of banking uncertainty on intangible investments, in the context that there has been a growing body of literature addressing the ramifications of aggregate uncertainty on overall investment or physical investment. Based on data from 619 non-financial listed companies and 40 commercial banks in Vietnam between 2007 and 2022, we perform empirical regression analysis using the dynamic generalized method of moments (GMM) estimations since firms’ investment behavior is significantly persistent. We make our investigations more relevant and comprehensive by considering banking uncertainty in Vietnam’s bank-based market and delving into the underlying mechanisms through which banking uncertainty impacts corporate investments. Our findings reveal that firms tend to curtail their intangible asset investments in response to higher banking sector uncertainty, albeit the magnitude of the impact is economically weak. Differently, banking uncertainty substantially constrains investments in tangible fixed assets. We further provide evidence on financial conditions (captured by the cost of debt, financing constraints, and firms’ bank debt) as a driving force underlying the relationship between banking uncertainty and tangible assets, but not intangible investments. Our results remain robust after considering alternative variables, accounting for potential structural disruptions arising from crises, and addressing potential endogeneity concerns.

## 1. Introduction

In recent years, the escalating uncertainty surrounding economic and financial aspects has underscored the imperative to comprehensively grasp their repercussions [1]. Within this line, numerous scholars have engaged in an exploration of the impact of uncertainty on corporate investments. Based on the theoretical frameworks of real options and financial friction, researchers posit that firms, when confronted with uncertainty, typically exhibit a propensity towards caution and conservatism, resulting in the deferral or reduction of investments while awaiting the resolution of uncertainties [2,3]. In contrast, it is argued that entrepreneurs can discern and take on investment opportunities amid conditions of uncertainty, ultimately reaping profits through resource integration [4]. Hence, uncertainty may indeed serve as a lucrative source of corporate gains. Overall, research addressing the interplay between corporate investment and uncertainty remains inconclusive.

This study investigates the influence of uncertainty on firm-level investment, encompassing not only the broader investment-uncertainty relationship explored in prior research but also delving into the specific connection between uncertainty and intangible investments. The following points trigger the motivation of this research issue. First, a multitude of earlier scholars have underscored the significance of uncertainty shocks in precipitating economic oscillations, primarily through their adverse effects on investment [5–8]. Nevertheless, compared to the extensive literature on aggregate or tangible (physical) investments, studies examining the effects of uncertainty on intangible investments remain relatively limited. This gap is significant given the growing importance of intangible capital. It is pertinent to acknowledge that the literature on endogenous growth has accorded greater emphasis on the role of intangible capital as a pivotal determinant of growth, productivity, and protracted macroeconomic undulations [9]. Furthermore, it is noteworthy that net investments in intangible capital have exceeded the proportion of net investments in tangible capital over the past decades [10], underscoring their increasing relevance in modern economies. Given these points, our study seeks to fill the present gap by focusing on how banking uncertainty impacts intangible asset investments, thereby contributing to a deeper understanding of how financial uncertainties influence firms’ allocation of resources between tangible and intangible assets.

Second, we need to consider that enterprises allocate their resources to financial assets, fixed assets, and innovation capital, and uncertainty can affect these investment categories differently. Firms with a higher share of intangible assets tend to rely more on internal funds for their investments, given the lower collateral value of intangible assets [11]. Intangible investments respond less to changes in corporate valuation and depreciate faster than tangible investments [12]. Thum-Thysen et al. [13] also note distinct responses of tangible and intangible assets to key determinants. Uncertainty and irreversibility can reduce investments, but this effect may vary depending on the investment type due to differing adjustment costs for tangibles and intangibles. Bloom [14] points out that intangibles exhibit greater irreversibility, making them potentially more susceptible to uncertainty. However, the strategic growth option theory suggests that firms may invest in intangible assets immediately to secure market share [15]. Furthermore, uncertainty increases financing costs and decreases future cash flows, leading to higher financing constraints and reduced investments [5]. As intangible investments are more often financed internally rather than externally, their sensitivity to external credit shocks, such as those induced by banking uncertainty, may be limited. In contrast, tangible assets, which are more reliant on external bank financing and typically serve as collateral, are more likely to be affected when uncertainty disrupts credit markets. Given this evidence, we hypothesize that banking-sector uncertainty may exert a stronger negative impact on tangible investments than on intangible ones, owing to these underlying differences in financing structures and sensitivity to credit conditions.

Third, while prior studies have examined the impact of uncertainties on corporate R&D and innovation investments—for instance, Wang et al. [16] find that policy and market uncertainties negatively affect R&D investments of Chinese listed firms.; Wen et al. [17] demonstrate that fiscal policy uncertainty reduces innovation investments in new energy enterprises; and Xu [18] shows that government policy uncertainty increases firms’ cost of capital, leading to decreased innovation—these studies primarily focus on R&D expenditures. However, R&D represents only a subset of intangible assets and does not fully capture the broader spectrum of intangible investments, which also include staff training, market development, and organizational efficiency. This distinction is crucial, as these other components may exhibit different responses to uncertainty. To address this gap, our study adopts a broader perspective, following the approach of Le et al. [19]. We focus on all identifiable intangibles with quantifiable values reported in balance sheets, extending beyond just R&D to include other vital intangible investments.

This study examines the impact of banking uncertainty on intangible investments in Vietnam. We acknowledge the work of Bontempi [20], which is closely similar to ours. Utilizing a sample of Italian firms, the prior author confirms that macroeconomic uncertainty plays a role in explaining investments in R&D and, to a lesser extent, non-R&D intangibles. Nevertheless, our study diverges from this work in several key dimensions. Most notably, Bontempi [20] primarily focuses on the uncertainty of firms’ future demand, relying exclusively on non-accounting data. Differently, we center our inquiry on uncertainty within the banking sector, a distinct source of uncertainty. In tangible and intangible investment scenarios, increased uncertainty can induce a “delay impact” on investment, as heightened uncertainty prompts firms to defer their expenditures. However, uncertainty within the banking sector may differ since it directly impacts the primary funding source for businesses, particularly in a context where intangible assets exhibit lesser dependence on external financing. In light of these predictions, an investigation into the influences of banking sector uncertainty on investments in intangible assets assumes pronounced significance. Furthermore, as pointed out by Lee [21], it is noteworthy that distinct uncertainty measures offer unique insights, underscoring the importance of considering various uncertainty indices.

Additionally, in an extension of the study by Bontempi [20], this paper further examines a crucial yet underexplored mechanism through which banking sector uncertainty impacts corporate intangible investments. Financing conditions play a pivotal role in determining the accessibility of external funding from the broader economic milieu, subsequently influencing firms’ investment decisions [13]. Uncertainty can elevate the risk premium in financial markets, escalate the cost of external financing, and curtail the availability of bank credit, rendering it arduous for firms to secure the necessary funds for their investment endeavors. Unlike the majority of theoretical and empirical investigations that explore the influence of uncertainty on investments within the context of irreversibility, our particular focus centers on comprehending the effects of banking sector-related uncertainty and elucidating the mechanisms through which it operates via external financing conditions.

Vietnam offers a pertinent case for our model testing, not only due to its underrepresentation in prior analyses but also because of its intriguing characteristics during the examined period. Given the limited development of the capital market, commercial banks in Vietnam play a central role in providing funds to the country’s financial markets, with corporate financing predominantly reliant on bank credit. This underscores the significant influence of banks and their close association with the activities of Vietnamese companies. Nevertheless, it is worth noting that Vietnamese banks have not yet attained a level of maturity in comparison to those in more developed markets [22]. It should be noted that as an emerging market, the level of uncertainty in Vietnam is more conspicuous than in developed markets [23], making banks in Vietnam suffer greater volatility than other banks in advanced economies [24]. Further, the uncertainty level in Vietnam in recent years may rise and become volatile substantially due to multiple forces, such as the global financial crisis, bad debt booms, various banking policy reforms, and the recent COVID-19 pandemic.

In light of Vietnam’s recent transition toward a “knowledge-based economy”, there has been a substantial upsurge in intangible investments by firms [25]. While the significance of such investments for enhancing competitive advantages and long-term yields of firms is widely acknowledged, research on intangible investments in the context of Vietnam remains scarce [19]. Furthermore, banks in more developed economies typically recognize and incorporate intangible assets of firms into their lending decisions, considering them as tangible items [26]. Nevertheless, it remains uncertain whether banks in Vietnam, operating within a less developed institutional framework marked by limited high-quality audits and weak copyright protection laws, share the same perspective on intangibles or view them as high-risk assets [19]. Moreover, the operational and investment risks faced by enterprises in Vietnam may surpass those encountered by their international counterparts. Banking institutions are pivotal in the risk mitigation strategies promulgated by governments [27]. The elevated level of uncertainty in the banking sector can engender a substantial augmentation in investment risk, necessitating a careful evaluation of this factor when enterprises deliberate their investment choices. Consequently, investigating the intersection of the banking system and intangible assets in Vietnam can yield valuable insights contributing to the existing body of literature.

We conduct our analysis using data from Vietnamese listed firms and the overall banking sector during 2007–2022. Inspired by Buch et al. [28], this study uses bank-level data to capture uncertainty in the banking sector. The uncertainty metric employed in the prior scholarly work operates on the premise that elevated levels of uncertainty are associated with reduced predictability of future outcomes. Consequently, a broader dispersion of perturbations affecting bank-level variables serves as an indicative marker of heightened uncertainty within the banking sector. We perform regressions using the dynamic generalized method of moments (GMM) estimations since firms’ investment behavior is persistent and significantly influenced by their prior-year activities. The study also ensures the robustness of findings by accounting for alternative variables, potential structural disruptions stemming from crises, and the potential endogeneity issue.

In contrast to the extensive body of literature addressing the ramifications of uncertainty on overall investment or physical investment, a notable gap exists in examining whether and to what extent uncertainty influences a specific subset of firm investments, particularly investments in intangible assets. Our study addresses this void by investigating the influence of banking sector uncertainty on investments in intangible assets. In this pursuit, our findings initially reveal that firms tend to curtail their intangible asset investments in response to an escalation in banking sector uncertainty, albeit the magnitude of the impact is somewhat economically weak. Intriguingly, our results elucidate that banking sector uncertainty is more strongly associated with investments in tangibles than intangibles. We make our investigation more relevant by considering banking uncertainty in the bank-based market of Vietnam.

Our investigation also delves into the underlying mechanisms through which banking uncertainty impacts corporate investments. In this vein, our contribution to the literature is evidenced by the role of external financing conditions on tangible asset investments, while we do not find such a significant role of these conditions in mediating the effect of banking uncertainty on investment in intangible assets. Elaborating further, we discern that as uncertainty levels surge, firms on average experience heightened financing costs and increased financial constraints, concomitant with a reduction in their reliance on bank debt. Consequently, amid heightened banking uncertainty, firms exhibit a reduced propensity to access external capital, culminating in diminished investment activities. Within this operational framework, intangible assets emerge as comparatively less susceptible to such fluctuations, primarily due to their proclivity for internal funding modalities as opposed to external capital sources. Taken together, in contexts characterized by elevated levels of banking uncertainty, it is advisable to accord heightened consideration to tangible investments within enterprises, as opposed to investments in corporate intangible assets. Distinctions between these asset categories are evident, with the requirement for external financial support being more pronounced for tangible fixed assets during periods of heightened uncertainty than their intangible counterparts.

## 2. Research methods

### 2.1. Data

This study requires financial data for assessing banking uncertainty and conducting firm investment regressions. We begin with a bank-level panel dataset comprising data from 40 commercial banks in Vietnam from 2007 to 2022, accounting for over 95% of the banking sector’s market shares in any sample year. Our sample retention involves observations with sufficient data for uncertainty calculations. Next, we compile a firm-level sample comprising 619 non-financial companies listed on the Ho Chi Minh City Stock Exchange (HOSE) and the Hanoi Stock Exchange (HNX). We exclude observations with missing essential data.

Our sample period commences in 2007 to coincide with the availability of audited financial information for both listed non-financial firms and commercial banks in Vietnam. To mitigate the impact of outliers, we employ winsorization at the 1st and 99th percentiles for firm-level variables. Data for firms and banks are sourced from the FiinPro database, and macroeconomic data are extracted from the International Financial Statistics repository.

### 2.2. Measurement of uncertainty

In this study, we employ the empirical framework that Buch et al. [28] put forth to estimate banking uncertainty. This estimation is achieved by analyzing the cross-sectional dispersion of shocks affecting bank-level variables. Consistent with the approach used by the prior authors, these critical bank-level variables cover (i) bank assets, represented by the growth in total assets, (ii) bank funding, assessed through the growth in short-term funding, and (iii) bank profitability, proxied by the return-on-assets ratio. To compute the dispersion of these shocks, we commence by determining bank-year-specific perturbations for each of the key variables, a process elucidated by the following regression model:


$$U_{i,t}\;=\;\alpha_{i}\;+{\;\beta }_{t}+{\;\epsilon }_{i,t\;}$$ (1)

where the symbol $U_{i,t}\;$represents the specific key bank-level variable of interest at year t of bank i, selected from the aforementioned three key variables. The model incorporates provisions for both bank-fixed effects ($\alpha_{i}$) and time-fixed effects ($\beta_{t}$). In this configuration, the regression residuals serve as indicators of the shocks to bank-level variables. Ultimately, the assessment of uncertainty within the entire banking sector (in year t), stemming from the dispersion observed across all bank-specific shocks, can be derived by computing the standard deviation of these residuals. An increase in the dispersion of shocks signifies a heightened level of uncertainty in banking. Recently, the methodology delineated above for formulating an uncertainty metric for the banking system has gained considerable attention in financial research [29–31].

### 2.3. Model specification

Our baseline model to test the link between uncertainty and intangible fixed assets is expressed as follows:


$${intang}_{i,t}\;={\;\alpha }_{\text{0}}\;+{\;\alpha_{\text{1}}\times intang}_{i,t–\text{1}}\;+\;\alpha_{\text{2}}\times {uncertainty}_{t–\text{1}}\;+\;\beta \times X_{i,t–\text{1}}\;+\;\gamma \times Z_{t–\text{1}}\;+{\;\alpha }_{i}\;+\;\epsilon_{i,t\;}$$ (2)

in which i and t correspond to the firm and year, respectively. The dependent variable, denoted as fixed intangible investment, is quantified as the ratio of intangible fixed assets to total assets of firms, in accordance with the approach employed in prior studies [12,32]. Detailed discussion on the reporting of intangible assets in Vietnam can be found in Le et al. [19]. Of notable significance is the inclusion of lagged intangible assets as an independent variable in equation 2, highlighting the dynamic in corporate investment behaviors. The principal independent variable of interest, *uncertainty*, is derived through the method elucidated in the preceding subsection.${\;\alpha }_{i}\;$denotes firm-fixed effects and $\epsilon_{i,t\;}$signifies error terms. It should be noted that an alternative inclusion of industry fixed effects still offers unchanged results, but we do not report them for brevity. We refrain from controlling for year fixed effects, as doing so might potentially eradicate all the effects of uncertainty, which are encapsulated by the level of country-specific banking market structure across sample years. Consistent with the existing literature on determinants of firm investment, particularly in the context of intangible investment [7,10,32], the model incorporates a set of control variables, encompassing both firm-level factors (denoted as X) and macroeconomic factors (indicated as Z). These control variables are: firm size (*size*), sales growth (*sale*), return on assets (*roa*), market value (*tobin*), leverage ratio (*lev*), policy rates (*rfr*), economic growth (*gdp*), and financial crisis (*crisis*). The precise definitions of these variables are outlined in Table 1.

**Table 1:** Summary statistics.

	Mean	SD	25th	75th	Calculations
intang	0.029	0.036	0.020	0.063	The ratio of intangible fixed assets to total assets
ppe	0.175	0.178	0.041	0.261	The ratio of tangible fixed assets to total assets
size	27.123	1.542	26.064	28.124	The natural logarithm of total assets
sale	0.202	0.559	−0.066	0.286	The growth of net sales
roa	0.063	0.062	0.019	0.094	The ratio of net profit to total average assets
tobin	1.120	0.545	0.807	1.254	The market value divided by book value (of total assets)
lev	0.489	0.228	0.262	0.674	The ratio of total liabilities to total assets
aunc	0.311	0.409	0.140	0.600	Banking uncertainty, based on the dispersion of asset shocks
func	0.454	0.519	0.167	0.998	Banking uncertainty, based on the dispersion of funding shocks
punc	0.005	0.001	0.004	0.007	Banking uncertainty, based on the dispersion of profit shocks
rfr	0.072	0.026	0.060	0.080	The refinancing rates announced by the central bank
gdp	0.059	0.015	0.053	0.068	The growth of GDP from year to year
crisis	0.136	0.342	0.000	1.000	A dummy that equals to one for the years 2007–2009

This table reports summary statistics for the main variables used in the analysis, based on a sample of 8,611 firm-year observations from 619 non-financial companies listed in Vietnam over the period 2007–2022.

Endogeneity poses a valid concern, as despite the inclusion of an extensive array of control variables, the potential presence of omitted variables that influence both banking uncertainty and firm investments cannot be entirely ruled out. To address endogeneity issues and emphasize the dynamic nature of investment practices, we adopt dynamic panel data techniques, specifically employing the two-step system GMM estimators. Within this framework, we consider the lagged dependent variable and firm-specific controls as predetermined or endogenous factors, recognizing that these firm-level variables lack strict exogeneity. Similarly, the designation of uncertainty as predetermined acknowledges the possibility that alterations in corporate investment behaviors may potentially induce changes in banking policy. Other macroeconomic variables are treated as exogenous factors. To ensure the validity of our GMM estimation, we subject our model to various specification tests, including the assessment of first- and second-order autocorrelation and the evaluation of overidentifying restrictions.

## 3. Results

### 3.1. Descriptive statistics

Table 1 presents a display of descriptive statistics for the examined variables. Over the sample period, it is evident that investments in tangible fixed assets significantly outweigh intangible investments, as the average proportion of intangible assets to total assets stands at 0.029, while the average share of tangible assets amounts to 0.175. This pattern is different from that of public firms in an advanced market [32]. In fact, despite the increasing trend in intangible investments shown recently in the literature, Vietnamese firms generally do not invest much in this type of asset [25]. The three uncertainty indices (aunc, func, and punc) capture shocks to bank assets, funding, and profits, respectively.*func* and *aunc* display considerable dispersion, with standard deviations that are close to or exceed their means, indicating substantial variability in funding and asset-related uncertainty over time. In contrast, *punc* has a smaller magnitude and lower absolute volatility, although the degree of variation remains meaningful relative to its narrow scale. These distinctions highlight the different dynamics represented by each uncertainty measure. Importantly, the descriptive patterns observed here are consistent with the work of Dang [29] for the Vietnamese banking market, which reinforces the validity of the uncertainty indices used in this study.

Additionally, the calculation of correlation coefficients among the variables has been conducted (although these results are unreported for brevity). The observed pairwise correlations among independent variables do not register high absolute terms, with all coefficients falling below the 0.5 threshold. This indicates that independent variables are not highly correlated, thereby wiping out concerns related to the multicollinearity.

### 3.2. Baseline results

Table 2 presents the baseline findings concerning the influence of uncertainty on intangible assets. The coefficient associated with the lagged dependent variable exhibits statistical significance, affirming the suitability of our dynamic model. Turning to the independent variable of primary interest, we observe that the coefficients for all alternative uncertainty metrics are negative and statistically significant at the 10% and 5% levels, implying that during periods of elevated banking uncertainty, firms tend to cut their investments in intangible assets. To quantify this effect, consider the estimation results reported in column 1 of Table 2, we can suggest that when banking uncertainty increases by one standard deviation (0.409), firms’ investments in intangible assets measured as the proportion of intangible investments decline by approximately 0.001 (−0.409*0.003), equivalent to 4.23% of the average intangible asset ratio (0.029). This pattern holds consistently when employing different measures of banking uncertainty as key independent variables. Hence, the uncertainty of the banking sector slightly reduces enterprises’ investment in intangible assets.

**Table 2:** Baseline results for uncertainty and intangible assets.

	Dependent variable: Intangible assets		
	(1)	(2)	(3)
Lagged dependent variable	0.801***	0.797***	0.793***
	(0.011)	(0.012)	(0.011)
aunc	−0.003*		
	(0.002)		
func		−0.005**	
		(0.002)	
punc			−0.639**
			(0.252)
size	0.001***	0.001***	0.001***
	(0.000)	(0.000)	(0.000)
sale	−0.002	−0.003	−0.002
	(0.002)	(0.002)	(0.002)
roa	−0.023**	−0.026***	−0.031***
	(0.009)	(0.009)	(0.009)
tobin	−0.015	−0.015	−0.018*
	(0.009)	(0.009)	(0.009)
lev	−0.021***	−0.024***	−0.024***
	(0.007)	(0.007)	(0.006)
rfr	0.066***	0.072***	0.057***
	(0.010)	(0.011)	(0.009)
gdp	−0.006	−0.013	−0.001
	(0.009)	(0.009)	(0.009)
crisis	0.001	−0.001	−0.004**
	(0.002)	(0.001)	(0.001)
Observations	7,988	7,988	7,988
Firms	623	623	623
Instruments	124	124	124
AR(1) test	0.000	0.000	0.000
AR(2) test	0.877	0.825	0.768
Hansen test	0.269	0.239	0.253

The table presents the estimations from the dynamic GMM model. Standard errors are between parentheses. * p < 0.1; ** p < 0.05; *** p < 0.01.

For comparative analysis, this study proceeds to examine the impact of uncertainty on tangible asset investments, presenting the results in Table 3. In columns 1–3, all uncertainty measures display negative and statistically significant coefficients (at the 1% and 5% levels), indicating a decrease in firms’ tangible assets under higher banking uncertainty. This effect also bears economic significance, wherein a one standard deviation escalation in uncertainty (0.519, column 2) is associated with a potential drop in the proportion of tangible assets by 0.060 (−0.519*0.115), accounting for 34.11% of the sample mean. All observed results consistently demonstrate this economically substantial influence. Furthermore, as evidenced in columns 4–6 of Table 3, the uncertainty index exhibits a negative and statistically significant relationship with the dependent variable of total fixed asset investments, reinforcing the notion that firms tend to reduce their real investments when confronted with heightened uncertainty.

**Table 3:** Decomposing total fixed assets.

	Dependent variable: Tangible assets (columns 1–3)			Dependent variable: Total fixed assets (columns 4–6)		
	(1)	(2)	(3)	(4)	(5)	(6)
Lagged dependent variable	0.859***	0.851***	0.856***	0.817***	0.836***	0.835***
	(0.016)	(0.017)	(0.016)	(0.013)	(0.013)	(0.013)
aunc	−0.151**			−0.178***		
	(0.073)			(0.051)		
func		−0.115**			−0.134**	
		(0.048)			(0.063)	
punc			−24.470***			−32.922***
			(2.260)			(6.979)
size	0.004**	0.004*	0.003	0.009***	0.002	0.002
	(0.002)	(0.002)	(0.002)	(0.002)	(0.002)	(0.002)
sale	−0.004**	−0.006***	−0.003**	0.001	−0.003**	−0.003*
	(0.001)	(0.001)	(0.001)	(0.004)	(0.002)	(0.002)
roa	0.094	0.260***	0.048	0.164**	0.132**	0.075
	(0.079)	(0.078)	(0.077)	(0.076)	(0.055)	(0.057)
tobin	0.107*	−0.005	0.111*	−0.224***	−0.005	0.038
	(0.062)	(0.066)	(0.061)	(0.058)	(0.052)	(0.050)
lev	0.137***	0.168***	0.107***	−0.060***	0.041	0.042
	(0.028)	(0.029)	(0.026)	(0.022)	(0.029)	(0.026)
rfr	0.148***	−0.032	0.121***	0.240***	0.151***	0.208***
	(0.043)	(0.042)	(0.042)	(0.050)	(0.043)	(0.047)
gdp	0.198***	0.150***	0.170***	0.286***	−0.041	0.245***
	(0.053)	(0.045)	(0.053)	(0.055)	(0.051)	(0.055)
crisis	−0.002	0.008	−0.018***	−0.006	−0.017***	−0.002
	(0.005)	(0.006)	(0.003)	(0.006)	(0.006)	(0.004)
Observations	7,988	7,988	7,988	7,988	7,988	7,988
Firms	623	623	623	623	623	623
Instruments	124	124	124	124	124	124
AR (1) test	0.000	0.000	0.000	0.000	0.000	0.000
AR (2) test	0.172	0.158	0.166	0.722	0.758	0.610
Hansen test	0.334	0.150	0.207	0.101	0.200	0.120

The table presents the estimations from the dynamic GMM model. Standard errors are between parentheses. * p < 0.1; ** p < 0.05; *** p < 0.01.

In summary, the results show that the impacts of banking uncertainty are stronger in the case of tangible assets compared to intangible assets, and such stronger effects tend to drive the overall firm investments under the pressure of uncertainty. We could propose a plausible rationale for this observation as follows. Intangible assets are typically financed through internal resources rather than external debt [13]. This reliance on internal funding buffers intangible investments from the fluctuations in credit availability that often accompany banking uncertainty. Since firms are less dependent on bank loans to finance these types of assets, the impact of banking uncertainty—manifested through tightened credit conditions [28,29]—is rather weak when it comes to intangible investments. This explains why the economic impact on intangible assets is relatively weak compared to tangible assets, which are more frequently financed through external debt and thus more sensitive to changes in credit availability. Additionally, the coefficients of the control variables, when statistically significant, align with the existing literature, although some may appear counterintuitive. Notably, the determinants of firm investments diverge between tangible and intangible assets. Given the study’s objectives, our regression analysis does not provide an exhaustive exploration of the control variables’ outcomes.

### 3.3. Robustness tests

While conducting our analysis, structural breaks or possible endogeneity may alter our estimates. In this subsection, we perform some further tests to wipe out these concerns and thus lend more strength to our findings.

Our research runs from 2007 to 2022, during which the economy and financial system were significantly impacted by macroeconomic shocks stemming from the 2007–2009 financial crisis and the 2020–2021 COVID-19 pandemic. These shocks likely changed the relationship between uncertainty and firm investments by affecting both factors. In light of this prediction, we perform additional subsample analyses by excluding these two periods characterized by substantial macro shocks. We present the subsample estimations in Table 4, focusing on both intangible assets and tangible investments as dependent variables. Interestingly, our findings remain unchanged even when the sample data is altered, underscoring the assertion that heightened banking uncertainty may engender a decline in various categories of corporate investments, with tangible assets being more affected than intangible investments.

**Table 4:** Robustness checks by excluding the crisis times (of 2007–2009 and 2020–2021).

	Dependent variable: Intangible assets (columns 1–3)			Dependent variable: Tangible assets (columns 4–6)		
	(1)	(2)	(3)	(4)	(5)	(6)
Lagged dependent variable	0.805***	0.802***	0.808***	0.871***	0.861***	0.849***
	(0.019)	(0.019)	(0.018)	(0.019)	(0.018)	(0.017)
aunc	−0.004*			−0.177**		
	(0.003)			(0.071)		
func		−0.005***			−0.152**	
		(0.002)			(0.072)	
punc			−0.069			−22.181***
			(0.312)			(2.299)
size	0.002***	0.002**	0.001***	0.001	0.001	0.007***
	(0.001)	(0.001)	(0.000)	(0.003)	(0.003)	(0.002)
sale	−0.001	−0.005	−0.003**	−0.003	−0.003*	−0.004**
	(0.003)	(0.003)	(0.001)	(0.002)	(0.002)	(0.002)
roa	−0.006	−0.007	−0.091***	0.109	0.113	0.256**
	(0.022)	(0.022)	(0.030)	(0.122)	(0.124)	(0.112)
tobin	−0.027*	−0.027*	−0.017	0.045	0.015	0.179**
	(0.014)	(0.014)	(0.010)	(0.110)	(0.114)	(0.091)
lev	−0.028***	−0.028***	−0.036***	0.080*	0.061	0.004
	(0.010)	(0.010)	(0.008)	(0.042)	(0.046)	(0.034)
rfr	0.047***	0.053***	0.021	0.136***	−0.031	0.138***
	(0.014)	(0.016)	(0.015)	(0.048)	(0.050)	(0.049)
gdp	−0.090	−0.121**	−0.077	0.148	0.432**	0.392**
	(0.056)	(0.058)	(0.047)	(0.164)	(0.197)	(0.172)
Observations	4,866	4,866	4,866	4,866	4,866	4,866
Firms	591	591	591	591	591	591
Instruments	81	81	81	81	81	81
AR (1) test	0.000	0.000	0.000	0.000	0.000	0.000
AR (2) test	0.845	0.765	0.713	0.497	0.492	0.459
Hansen test	0.435	0.496	0.384	0.336	0.137	0.220

The table presents the estimations from the dynamic GMM model. Standard errors are between parentheses. * p < 0.1; ** p < 0.05; *** p < 0.01.

Although the likelihood of reverse causality between uncertainty at the country level and corporate investments at the firm level is minimal, we still employ a further econometric strategy to address potential endogeneity concerns. Specifically, we employ the Economic Policy Uncertainty (EPU) index of China, which happens to be Vietnam’s largest trading partner, as an instrumental variable in a two-stage least squares (2SLS) regression [7,33]. This choice of instrumental variable is substantiated by its direct correlation with Vietnamese uncertainty, while lacking any apparent rationale to assert a direct influence on the investments of Vietnamese firms. As such, it meets the criteria for a valid instrumental variable. We run estimations employing the strategy mentioned above. Examining the regression results as presented in Table 5, it becomes evident that the coefficients associated with the measures of uncertainty exhibit statistical significance and maintain consistent signs in both models focusing on intangible and tangible assets. This reaffirms the robustness of our primary findings. To conserve space, the first-stage regression results are provided in S1 Table.

**Table 5:** Further endogeneity treatment.

	Dependent variable: Intangible assets (columns 1–3)			Dependent variable: Tangible assets (columns 4–6)		
	(1)	(2)	(3)	(4)	(5)	(6)
aunc	−0.006**			−0.321***		
	(0.003)			(0.105)		
func		−0.007***			−0.222***	
		(0.002)			(0.070)	
punc			−0.134**			−13.065***
			(0.063)			(4.120)
size	0.001**	0.001*	0.001	0.013***	0.012***	0.010***
	(0.001)	(0.001)	(0.001)	(0.003)	(0.003)	(0.002)
sale	−0.001***	−0.002***	−0.002***	−0.003*	−0.001	−0.002
	(0.000)	(0.001)	(0.000)	(0.002)	(0.002)	(0.002)
roa	−0.036***	−0.038***	−0.032***	0.128***	0.138***	0.103***
	(0.008)	(0.009)	(0.008)	(0.033)	(0.035)	(0.032)
tobin	0.002	−0.001	0.005	−0.018	−0.018	0.015
	(0.003)	(0.003)	(0.003)	(0.018)	(0.019)	(0.018)
lev	−0.010***	−0.011***	−0.008***	0.039***	0.035***	0.054***
	(0.003)	(0.003)	(0.003)	(0.011)	(0.012)	(0.010)
rfr	0.007	0.182**	0.051***	0.003	0.936**	0.313***
	(0.019)	(0.093)	(0.014)	(0.075)	(0.371)	(0.057)
gdp	0.026	−0.145***	−0.002	0.386***	1.023***	0.237**
	(0.019)	(0.052)	(0.024)	(0.075)	(0.207)	(0.096)
crisis	−0.010**	−0.052**	−0.0001	−0.049***	−0.271***	0.003
	(0.005)	(0.023)	(0.001)	(0.019)	(0.091)	(0.004)
Observations	7,988	7,988	7,988	7,988	7,988	7,988
Underidentification test	219.421***	14.631***	1407.927***	219.421***	14.631***	1407.927***
Weak identification test	226.526	14.641	1780.754	226.526	14.641	1780.754

The table presents the estimations from the IV-2SLS approach. To conserve space, only the second-stage estimates are displayed here, while the first-stage regression results are provided in the Appendix. Standard errors are between parentheses. * p < 0.1; ** p < 0.05; *** p < 0.01.

### 3.4. Mechanism analysis

Uncertainty potentially impedes firm investment by influencing financing conditions. Extensive research indicates that heightened uncertainty prompts banks and other financial intermediaries to adopt a more cautious stance, mitigating risk exposure through elevated lending rates and reduced credit availability [28,31,34,35]. Consequently, corporate financing options become scarcer, and financing costs rise. In the context of our study, heightened uncertainty within the banking sector is likely to curtail the availability of bank loans, elevate financing costs, and intensify financial constraints. To empirically check these predictions, we now examine the channels through which banking uncertainty exerts its influence on firm investments. Building on the arguments above, we focus on three potential mediators: the cost of debt, financing constraints, and firms’ bank debt.

To examine the cost-of-debt channel through which banking uncertainty affects firm investments, we initially assess the relationship between the cost of debt and uncertainty. In Table 6, we estimate the sensitivity of the cost of debt to variations in banking uncertainty while controlling for key factors that directly affect the cost of debt, as advised by existing literature [36,37]. Using the ratio of interest expenses to corporate debt as the dependent variable, our analysis reveals that the coefficients associated with uncertainty are consistently positive and statistically significant in the alternative models, indicating that the cost of debt increases with heightened banking uncertainty. In this analysis, banking uncertainty is measured at time t–1, while the cost of debt is measured at time t, reflecting the assumption that credit conditions respond to uncertainty observed in the previous year.

**Table 6:** Cost of debt under uncertainty.

	Dependent variable: cost of debt		
	(1)	(2)	(3)
Lagged dependent variable	−0.408***	−0.421***	−0.412***
	(0.010)	(0.010)	(0.010)
aunc	0.158***		
	(0.010)		
func		0.075***	
		(0.008)	
punc			6.389***
			(0.658)
size	−0.007***	−0.008***	−0.009***
	(0.001)	(0.001)	(0.001)
sale	0.001	0.003**	0.002
	(0.001)	(0.001)	(0.001)
roa	−0.161***	−0.147***	−0.141***
	(0.024)	(0.023)	(0.023)
lev	−0.023***	−0.020**	−0.016**
	(0.008)	(0.008)	(0.008)
state	0.005*	0.005*	0.005
	(0.003)	(0.003)	(0.003)
rfr	0.985***	0.836***	1.074***
	(0.041)	(0.045)	(0.043)
gdp	−0.580***	−0.402***	−0.456***
	(0.052)	(0.051)	(0.055)
crisis	−0.058***	−0.083***	−0.024***
	(0.005)	(0.008)	(0.004)
Observations	7,988	7,988	7,988
Firms	623	623	623
Instruments	137	137	137
AR (1) test	0.000	0.000	0.000
AR (2) test	0.375	0.426	0.583
Hansen test	0.231	0.172	0.236

The table presents the estimations from the dynamic GMM model. *state* is the percentage of capital held by the government. Other variables are the same as defined in Table 1. Standard errors are between parentheses. * p < 0.1; ** p < 0.05; *** p < 0.01.

Next, to thoroughly verify the cost of debt channel, Table 7 presents the regressions referring to the effects of uncertainty and the cost of debt on investment in tangible and intangible assets. The estimates, generated using the dynamic GMM estimator for the uncertainty variables, closely resemble the previously obtained results, thereby corroborating our earlier findings. In these regressions, we retain the following timing structure: uncertainty is measured at time t–1, while both the cost of debt and investment are measured at time t. In alignment with the expectations stemming from the cost of debt channel, we observe that, on average, as uncertainty in the banking sector intensifies, the cost of debt rises, leading to reduced investments in tangible assets. This is illustrated by negative and statistically significant coefficients associated with the cost of debt variable in columns 4–6 of Table 7. However, our results do not establish a consistent and significant association between financing costs and investments in intangible assets (columns 1–3), suggesting that the cost of debt channel does not effectively elucidate the impact of banking uncertainty on intangible asset investments.

**Table 7:** Mechanism tests for the cost of debt.

	Dependent variable: Intangible assets (columns 1–3)			Dependent variable: Tangible assets (columns 4–6)		
	(1)	(2)	(3)	(4)	(5)	(6)
Lagged dependent variable	0.844***	0.844***	0.843***	0.871***	0.870***	0.863***
	(0.014)	(0.014)	(0.014)	(0.014)	(0.013)	(0.013)
aunc	−0.006***			−0.194**		
	(0.001)			(0.091)		
func		−0.004**			−0.110***	
		(0.002)			(0.037)	
punc			−0.561*			−12.666***
			(0.292)			(2.049)
cost of debt	−0.091*	0.042	−0.063	−0.056***	−0.055***	−0.054***
	(0.050)	(0.036)	(0.051)	(0.009)	(0.009)	(0.009)
size	0.001	0.003	0.001	0.005**	0.006***	0.008***
	(0.001)	(0.002)	(0.002)	(0.002)	(0.002)	(0.002)
sale	−0.002	−0.001	−0.005	−0.003**	−0.004***	−0.003***
	(0.003)	(0.003)	(0.003)	(0.001)	(0.001)	(0.001)
roa	−0.013	−0.014	−0.011	0.208***	0.271***	0.231***
	(0.010)	(0.010)	(0.010)	(0.080)	(0.074)	(0.079)
tobin	−0.016**	−0.015*	−0.015*	−0.115	−0.154**	−0.168**
	(0.008)	(0.008)	(0.008)	(0.073)	(0.069)	(0.069)
lev	0.003	0.001	0.002	0.004	0.028	−0.024
	(0.006)	(0.005)	(0.005)	(0.028)	(0.027)	(0.026)
rfr	0.013	0.011	−0.006	0.245***	0.025	0.243***
	(0.015)	(0.015)	(0.015)	(0.059)	(0.043)	(0.059)
gdp	−0.015*	−0.019**	−0.022***	0.195***	0.089*	0.186**
	(0.008)	(0.008)	(0.007)	(0.074)	(0.049)	(0.073)
crisis	−0.122***	−0.152***	−0.178***	0.007	0.008	−0.006
	(0.046)	(0.045)	(0.041)	(0.006)	(0.007)	(0.004)
Observations	7,988	7,988	7,988	7,988	7,988	7,988
Firms	623	623	623	623	623	623
Instruments	124	124	124	124	124	124
AR (1) test	0.000	0.000	0.000	0.000	0.000	0.000
AR (2) test	0.807	0.764	0.785	0.708	0.505	0.687
Hansen test	0.112	0.192	0.133	0.514	0.273	0.471

The table presents the estimations from the dynamic GMM model. Standard errors are between parentheses. * p < 0.1; ** p < 0.05; *** p < 0.01.

Following the same procedure as exhibited above, Table 8 displays the results of the effect of uncertainty on financing constraints, based on regressions with controls in line with previous studies [38,39]. We employ two proxy variables, the *KZ* index and *WW* index, derived from the methodologies proposed by Kaplan and Zingales [40] and Whited and Wu [41], respectively, to gauge the extent of financing constraints. Higher values on these proxies denote more pronounced financing constraints. Our analysis in Table 8 reveals that the coefficients associated with uncertainty are statistically significant and positive, indicating that firms encounter greater financing constraints in periods of increased banking uncertainty.

**Table 8:** Corporate financing constraints under uncertainty.

	Dependent variable: KZ index			Dependent variable: WW index		
	(1)	(2)	(3)	(4)	(5)	(6)
Lagged dependent variable	0.432***	0.446***	0.434***	0.202***	0.206***	0.217***
	(0.013)	(0.013)	(0.012)	(0.022)	(0.022)	(0.022)
aunc	44.913***			0.025***		
	(11.069)			(0.006)		
func		24.091***			0.026***	
		(5.753)			(0.009)	
punc			549.579**			1.748***
			(263.006)			(0.383)
size	−0.870***	−0.738***	−0.640***	−0.033***	−0.032***	−0.032***
	(0.192)	(0.184)	(0.180)	(0.001)	(0.001)	(0.001)
cash	1.950	0.336	3.410*	−0.026***	−0.023***	−0.023***
	(1.977)	(2.046)	(1.983)	(0.006)	(0.006)	(0.006)
ros	23.804***	21.191***	25.920***	−0.004	−0.017	−0.007
	(3.938)	(4.031)	(4.070)	(0.014)	(0.014)	(0.014)
state	0.113	−0.143	−0.127	−0.006***	−0.006***	−0.006***
	(0.395)	(0.387)	(0.390)	(0.001)	(0.001)	(0.001)
lev	3.079***	2.478**	3.408***	0.011***	0.008**	0.011***
	(1.135)	(1.160)	(1.140)	(0.003)	(0.003)	(0.003)
tobin	−1.325***	−1.151***	−1.656***	−0.019***	−0.018***	−0.018***
	(0.391)	(0.399)	(0.399)	(0.001)	(0.001)	(0.001)
rfr	57.761***	100.855***	38.681**	0.046**	0.047	0.013
	(15.505)	(23.455)	(16.162)	(0.018)	(0.038)	(0.017)
gdp	49.190***	33.351*	24.751	−0.274***	−0.102***	−0.260***
	(18.670)	(18.677)	(18.367)	(0.026)	(0.032)	(0.027)
crisis	4.272	5.698	−5.920**	−0.012***	0.015*	−0.016***
	(3.529)	(4.123)	(2.969)	(0.002)	(0.009)	(0.001)
Observations	7,988	7,988	7,988	7,988	7,988	7,988
Firms	623	623	623	623	623	623
Instruments	111	111	111	111	111	111
AR (1) test	0.000	0.000	0.000	0.000	0.000	0.000
AR (2) test	0.684	0.423	0.229	0.674	0.793	0.586
Hansen test	0.172	0.170	0.121	0.200	0.214	0.315

The table presents the estimations from the dynamic GMM model. The regression model has new controls: cash and cash equivalents scaled by total assets *– cash*, the percenstage of capital held by the government *– state,* and net income divided by net sales *– ros*. Other variables are the same as defined in Table 1. Standard errors are between parentheses. * p < 0.1; ** p < 0.05; *** p < 0.01.

We then completely ascertain the role of financing constraints by exploring the effects of financial constraints and uncertainty on firm investments. As presented in Tables 9 and 10, where we examine investments in different asset types, we observe that both proxy variables for financing constraints exhibit a statistically significant negative correlation with investments in tangible assets (columns 4–6 of two tables). However, their relationship with investments in intangible assets is mixed and statistically insignificant (columns 1–3). Consequently, we can conclude that financing constraints serve as a channel through which uncertainty impacts investments in tangible assets, while this mechanism appears to play no role in explaining investments in intangible assets.

**Table 9:** Mechanism tests for financing constraints by KZ index.

	Dependent variable: Intangible assets (columns 1–3)			Dependent variable: Tangible assets (columns 4–6)		
	(1)	(2)	(3)	(4)	(5)	(6)
Lagged dependent variable	0.806***	0.802***	0.800***	0.884***	0.877***	0.881***
	(0.011)	(0.011)	(0.011)	(0.017)	(0.017)	(0.016)
aunc	−0.004***			−0.122**		
	(0.001)			(0.052)		
func		−0.005**			−0.103***	
		(0.002)			(0.025)	
punc			−0.596**			−13.700***
			(0.269)			(2.397)
KZ index	−0.014	0.006	−0.001	−0.002***	−0.002***	−0.002***
	(0.010)	(0.013)	(0.000)	(0.001)	(0.001)	(0.001)
size	0.002***	0.002***	0.002***	0.008***	0.009***	0.006***
	(0.000)	(0.000)	(0.000)	(0.002)	(0.003)	(0.002)
sale	0.013	0.001	0.009	−0.007***	−0.010***	−0.006***
	(0.013)	(0.000)	(0.012)	(0.002)	(0.002)	(0.002)
roa	−0.029***	−0.033***	−0.035***	0.226***	0.428***	0.186**
	(0.009)	(0.009)	(0.009)	(0.084)	(0.083)	(0.084)
tobin	−0.031***	−0.030***	−0.031***	0.078	−0.040	0.083
	(0.010)	(0.011)	(0.011)	(0.063)	(0.069)	(0.062)
lev	−0.022***	−0.025***	−0.024***	0.150***	0.200***	0.124***
	(0.006)	(0.006)	(0.006)	(0.030)	(0.031)	(0.028)
rfr	0.069***	0.076***	0.056***	0.141***	−0.015	0.126***
	(0.010)	(0.011)	(0.010)	(0.044)	(0.043)	(0.043)
gdp	−0.001	−0.021**	−0.008	0.182***	0.141***	0.152***
	(0.000)	(0.010)	(0.011)	(0.058)	(0.046)	(0.057)
crisis	0.001	−0.001	−0.004**	−0.010*	0.002	−0.023***
	(0.002)	(0.001)	(0.002)	(0.006)	(0.006)	(0.004)
Observations	7,988	7,988	7,988	7,988	7,988	7,988
Firms	623	623	623	623	623	623
Instruments	124	124	124	124	124	124
AR (1) test	0.000	0.000	0.000	0.000	0.000	0.000
AR (2) test	0.625	0.565	0.536	0.372	0.355	0.614
Hansen test	0.393	0.327	0.369	0.202	0.440	0.166

The table presents the estimations from the dynamic GMM model. Standard errors are between parentheses. * p < 0.1; ** p < 0.05; *** p < 0.01.

**Table 10:** Mechanism tests for financing constraints by WW index.

	Dependent variable: Intangible assets (columns 1–3)			Dependent variable: Tangible assets (columns 4–6)		
	(1)	(2)	(3)	(4)	(5)	(6)
Lagged dependent variable	0.800***	0.792***	0.793***	0.858***	0.853***	0.855***
	(0.012)	(0.013)	(0.012)	(0.016)	(0.017)	(0.016)
aunc	−0.008***			−0.195***		
	(0.001)			(0.045)		
func		−0.005***			−0.170***	
		(0.002)			(0.044)	
punc			−0.625**			−15.269***
			(0.314)			(4.372)
WW index	−0.030	−0.021	−0.029	−0.318***	−0.215**	−0.378***
	(0.020)	(0.015)	(0.020)	(0.101)	(0.095)	(0.099)
size	0.001	0.001	0.001	0.008*	0.004	0.011***
	(0.001)	(0.001)	(0.001)	(0.004)	(0.004)	(0.004)
sale	0.001	−0.001	0.001	−0.010***	−0.010***	−0.011***
	(0.000)	(0.001)	(0.000)	(0.002)	(0.002)	(0.002)
roa	−0.002	0.002	−0.002	0.163**	0.307***	0.128
	(0.013)	(0.012)	(0.013)	(0.081)	(0.079)	(0.079)
tobin	−0.022**	−0.030***	−0.024**	0.104*	−0.009	0.113*
	(0.011)	(0.010)	(0.011)	(0.063)	(0.068)	(0.063)
lev	−0.025***	−0.028***	−0.027***	0.165***	0.186***	0.144***
	(0.007)	(0.007)	(0.007)	(0.030)	(0.031)	(0.028)
rfr	0.069***	0.028	0.060***	0.121***	−0.012	0.096**
	(0.011)	(0.028)	(0.011)	(0.043)	(0.042)	(0.043)
gdp	−0.031***	−0.034***	−0.039***	0.285***	0.129***	0.268***
	(0.011)	(0.010)	(0.011)	(0.058)	(0.046)	(0.057)
crisis	0.001	−0.020***	−0.003**	−0.002	0.009	−0.015***
	(0.002)	(0.005)	(0.002)	(0.005)	(0.006)	(0.003)
Observations	7,988	7,988	7,988	7,988	7,988	7,988
Firms	623	623	623	623	623	623
Instruments	124	124	124	124	124	124
AR (1) test	0.000	0.000	0.000	0.000	0.000	0.000
AR (2) test	0.853	0.782	0.758	0.400	0.266	0.155
Hansen test	0.355	0.292	0.356	0.167	0.163	0.249

The table presents the estimations from the dynamic GMM model. Standard errors are between parentheses. * p < 0.1; ** p < 0.05; *** p < 0.01.

Finally, we repeat the estimation stages with bank debt as a mediator, captured by the share of bank debt in firms’ total assets. In the first step to test the impact of uncertainty on bank debt, we run our model specification as inspired by prior authors [42,43] and then report results in Table 11. We can see that the uncertainty coefficient is all negative and significant, indicating that uncertainty reduces bank debt held by firms. In the second step, with results presented in Table 12, we find that while bank debt positively influences investments in physical assets, it does not appear to be a critical determinant of investments in intangible assets. These estimates collectively suggest that firms curtail their investments in physical assets during uncertain periods due to a decrease in bank loans, but this decrease does not constitute a significant obstacle to intangible asset investments for enterprises.

**Table 11:** Firms’ bank debt under uncertainty.

	Dependent variable: Bank debt		
	(1)	(2)	(3)
Lagged dependent variable	0.576***	0.592***	0.578***
	(0.023)	(0.020)	(0.022)
aunc	−0.088***		
	(0.021)		
func		−0.069***	
		(0.026)	
punc			−2.837***
			(1.078)
size	0.010***	0.010***	0.010***
	(0.001)	(0.001)	(0.001)
cash	−0.062***	−0.068***	−0.065***
	(0.016)	(0.016)	(0.016)
ros	0.069**	0.035	0.076***
	(0.027)	(0.027)	(0.027)
state	−0.006	−0.004	−0.006
	(0.004)	(0.004)	(0.004)
lev	0.090***	0.078***	0.091***
	(0.014)	(0.013)	(0.013)
tobin	−0.009***	−0.003	−0.009***
	(0.003)	(0.003)	(0.003)
rfr	0.206***	0.117	0.336***
	(0.055)	(0.112)	(0.050)
gdp	0.148	0.875***	0.108
	(0.096)	(0.122)	(0.099)
crisis	−0.004	−0.052*	0.014***
	(0.005)	(0.028)	(0.004)
Observations	7,988	7,988	7,988
Firms	623	623	623
Instruments	111	111	111
AR (1) test	0.000	0.000	0.000
AR (2) test	0.726	0.435	0.817
Hansen test	0.246	0.129	0.289

The table presents the estimations from the dynamic GMM model. The regression model has new controls: cash and cash equivalents scaled by total assets *– cash*, the percentage of capital held by the government *– state,* and net income divided by net sales *– ros*. Other variables are the same as defined in Table 1. Standard errors are between parentheses. * p < 0.1; ** p < 0.05; *** p < 0.01.

**Table 12:** Mechanism tests for financing constraints by the reduction of bank debt.

	Dependent variable: Intangible assets (columns 1–3)			Dependent variable: Tangible assets (columns 4–6)		
	(1)	(2)	(3)	(4)	(5)	(6)
Lagged dependent variable	0.827***	0.827***	0.823***	0.854***	0.829***	0.841***
	(0.009)	(0.009)	(0.009)	(0.015)	(0.016)	(0.015)
aunc	−0.012***			−0.210***		
	(0.004)			(0.049)		
func		−0.006**			−0.122**	
		(0.002)			(0.052)	
punc			−0.559**			−20.749***
			(0.239)			(2.025)
bank debt	−0.002	−0.007	−0.002	0.035*	0.127***	0.040*
	(0.009)	(0.009)	(0.009)	(0.019)	(0.022)	(0.021)
size	0.001	0.001**	0.001**	0.003***	0.001	0.005***
	(0.000)	(0.000)	(0.000)	(0.001)	(0.002)	(0.001)
sale	−0.001	−0.001	−0.001	−0.002**	−0.004***	−0.004***
	(0.000)	(0.000)	(0.000)	(0.001)	(0.001)	(0.001)
roa	−0.011**	−0.018***	−0.013***	0.089	0.270***	0.229***
	(0.005)	(0.005)	(0.005)	(0.069)	(0.074)	(0.069)
tobin	0.001	0.002	−0.001	−0.016	−0.114*	−0.138**
	(0.008)	(0.008)	(0.008)	(0.054)	(0.062)	(0.057)
rfr	0.061***	0.066***	0.047***	0.194***	0.013	0.078*
	(0.009)	(0.011)	(0.009)	(0.043)	(0.038)	(0.047)
gdp	−0.008	−0.015**	−0.016**	0.178***	0.120***	0.095**
	(0.007)	(0.006)	(0.006)	(0.054)	(0.045)	(0.045)
crisis	−0.001	−0.002**	−0.005***	−0.003	−0.001	−0.008*
	(0.001)	(0.001)	(0.001)	(0.005)	(0.006)	(0.005)
Observations	7,988	7,988	7,988	7,988	7,988	7,988
Firms	623	623	623	623	623	623
Instruments	124	124	124	124	124	124
AR (1) test	0.000	0.000	0.000	0.000	0.000	0.000
AR (2) test	0.954	0.882	0.823	0.162	0.189	0.139
Hansen test	0.325	0.277	0.269	0.300	0.172	0.109

The table presents the estimations from the dynamic GMM model. Standard errors are between parentheses. * p < 0.1; ** p < 0.05; *** p < 0.01.

In sum, the findings show that banking uncertainty is negatively associated with corporate investment through at least three channels related to financial conditions: the cost of debt, financing constraints, and corporate bank debt. Interestingly, these channels only work for firms’ investment decisions in tangible assets, while similar routes for intangible asset investments are not observed. We discuss that when banking uncertainty increases, it may hurt the credit supply of banks – an essential source of external funding [28,29], which is comparably more critical for investment in tangibles compared to intangibles [13]. This differential effect can be further understood through the lens of the pecking order theory [44], which posits that firms prioritize internal over external financing to avoid adverse selection costs associated with raising external capital. Given that intangible investments are often difficult to collateralize and assess externally, firms may strategically rely on internal funds to finance these projects, particularly during periods of heightened uncertainty when the cost of external finance rises and information asymmetries are amplified [4,5].

## 4. Conclusions

While economic and financial uncertainty have been scrutinized extensively from various angles, one facet that remains underexplored is the impact of such uncertainties on firms’ intangible investments. Addressing this gap, our study contributes to the broader literature by examining the relationship between banking uncertainty and corporate intangible investments. In light of Vietnam’s status as a bank-based country, which has recently transitioned towards a “knowledge-based economy”, we have an appropriate setting for investigating the nexus between banking uncertainty and intangible investments. Employing data from 2007 to 2022, this research provides empirical insights that lead to the following conclusions: i) banking uncertainty exerts a limited influence on reduced investment in intangible fixed assets, ii) banking uncertainty substantially constrains investments in tangible fixed assets, and iii) the impact of banking uncertainty on tangible fixed assets operates through the channels of the financing costs, financial constraints, and bank debt of firms, whereas these mechanisms do not significantly mediate the impact of uncertainty on intangible fixed assets.

The empirical findings derived from this study yield several implications. Given the relatively limited sensitivity of intangible asset investments to banking sector volatility and the minor role of external finance in driving such investments, our findings suggest that credit-based interventions may be less effective for stimulating intangible investment during periods of heightened banking uncertainty. This does not imply that industries heavily reliant on intangible assets, such as technology, pharmaceuticals, and creative industries, are unimportant or should receive less policy attention. Rather, the evidence indicates that these industries are less adversely affected through the bank-credit channel in uncertain times, and thus may require policy instruments other than credit expansion to be effectively supported. Accordingly, policymakers aiming to strengthen these strategically vital sectors should consider complementing financial support with alternative mechanisms that better align with the capital structure and financing behavior of intangible-intensive industries.

In contrast, policy initiatives should be devised to facilitate enterprises’ access to external funding during periods characterized by banking sector uncertainty, with a specific focus on bolstering investments in tangible assets. Given that banking uncertainty affects tangible assets primarily through financing costs, financial constraints, and bank debt, policymakers should implement strategies to alleviate these constraints, such as enhancing access to credit for firms, especially during periods of uncertainty. This could involve creating government-backed loan programs, offering interest rate subsidies, or establishing credit guarantee schemes to reduce the cost of borrowing and ensure that firms can continue to invest in necessary tangible assets. Alternatively, policymakers should also focus on measures that reduce volatility in the banking sector, such as strengthening regulatory frameworks and enhancing the transparency of monetary policies. Besides, it is essential to promote alternative financing sources beyond traditional bank loans to mitigate the impact of banking uncertainty on tangible investments.

Our study’s focus on Vietnam, with its distinct economic and financial landscape, raises concerns about the generalizability of our results to other contexts. We recognize that Vietnam’s banking system and corporate investment behaviors may differ significantly from those in other emerging markets or developed economies. Another limitation of this study is the absence of a precise explanation for how banking uncertainty affects firms’ investments in intangible assets, primarily due to its minor impact. Investigating the economic mechanism necessitates in-depth analysis using firm-level data, necessarily motivated by a significant uncertainty impact. Furthermore, while we define intangible investments broadly to include components such as staff training, market development, and R&D, our empirical analysis relies on balance sheet-reported data, which inherently excludes informal intangibles such as knowledge spillovers and informal learning. This limitation underscores the need for improved and more granular data. Additionally, the study incorporates the weighting of intangible and tangible assets within total assets as dependent variables; however, it does not explicitly consider investments in cash performed each year due to data limitations. In summary, these arguments proffer promising avenues for future scholarly work that conducts comparative analyses across different regions with data improvement, which would help to validate and extend the applicability of our findings.

## Supporting information

S1 Table First-stage estimation results of IV-2SLS regressions. (DOCX)
