# Supplementary material for: The impact of banking uncertainty on firm investment: A look into intangible assets
Source: PLoS One. 2026 Jan 23;21(1):e0340913. doi: 10.1371/journal.pone.0340913 (PMC12829826; doi:10.1371/journal.pone.0340913)
Supplement: S1 Table — (DOCX) [file pone.0340913.s001.docx]

S1 Table. First-stage estimation results of IV-2SLS regressions

|  | (1) | (2) | (3) |
| --- | --- | --- | --- |
|  | aunc | func | punc |
| EPU | 0.025*** | 0.050*** | 0.001*** |
|  | (0.002) | (0.005) | (0.000) |
| size | -0.013*** | -0.041*** | 0.000 |
|  | (0.002) | (0.005) | (0.000) |
| sale | 0.003** | 0.017*** | 0.000 |
|  | (0.001) | (0.004) | (0.000) |
| roa | -0.004 | -0.048 | 0.001*** |
|  | (0.020) | (0.062) | (0.000) |
| tobin | 0.004** | 0.015** | 0.000** |
|  | (0.002) | (0.006) | (0.000) |
| lev | 0.003 | -0.005 | 0.000 |
|  | (0.006) | (0.020) | (0.000) |
| rfr | 0.884*** | 4.540*** | 0.002*** |
|  | (0.029) | (0.090) | (0.000) |
| gdp | 0.025 | -0.836*** | -0.011*** |
|  | (0.047) | (0.143) | (0.001) |
| crisis | 0.221*** | 0.409*** | 0.001*** |
|  | (0.002) | (0.007) | (0.000) |
| Observations | 7,988 | 7,988 | 7,988 |
| R-squared | 0.537 | 0.505 | 0.496 |
| This table reports the first-stage regression results of the IV-2SLS estimation used in the robustness analysis. The dependent variable in each column is the corresponding measure of banking uncertainty. The instrument employed is China’s Economic Policy Uncertainty (EPU) index. The strong and statistically significant coefficients on the instrument support its relevance. Full variable definitions are provided in Table 1 of the main text. | | | |
